# Fluphenazine Reduces Proteotoxicity in *C. elegans* and Mammalian Models of Alpha-1-Antitrypsin Deficiency

**DOI:** 10.1371/journal.pone.0087260

**Published:** 2014-01-31

**Authors:** Jie Li, Stephen C. Pak, Linda P. O’Reilly, Joshua A. Benson, Yan Wang, Tunda Hidvegi, Pamela Hale, Christine Dippold, Michael Ewing, Gary A. Silverman, David H. Perlmutter

**Affiliations:** 1 Department of Pediatrics, University of Pittsburgh School of Medicine, Pittsburgh, Pennsylvania, United States of America; 2 Department of Cell Biology and Physiology, University of Pittsburgh School of Medicine, Pittsburgh, Pennsylvania, United States of America; 3 Children’s Hospital of Pittsburgh of University of Pittsburgh Medical Center, Pittsburgh, Pennsylvania, United States of America; Centro de Investigación en Medicina Aplicada (CIMA), Spain

## Abstract

The classical form of α1-antitrypsin deficiency (ATD) is associated with hepatic fibrosis and hepatocellular carcinoma. It is caused by the proteotoxic effect of a mutant secretory protein that aberrantly accumulates in the endoplasmic reticulum of liver cells. Recently we developed a model of this deficiency in *C. Elegans* and adapted it for high-content drug screening using an automated, image-based array scanning. Screening of the Library of Pharmacologically Active Compounds identified fluphenazine (Flu) among several other compounds as a drug which reduced intracellular accumulation of mutant α1-antitrypsin Z (ATZ). Because it is representative of the phenothiazine drug class that appears to have autophagy enhancer properties in addition to mood stabilizing activity, and can be relatively easily re-purposed, we further investigated its effects on mutant ATZ. The results indicate that Flu reverses the phenotypic effects of ATZ accumulation in the *C. elegans* model of ATD at doses which increase the number of autophagosomes *in vivo*. Furthermore, in nanomolar concentrations, Flu enhances the rate of intracellular degradation of ATZ and reduces the cellular ATZ load in mammalian cell line models. In the PiZ mouse model Flu reduces the accumulation of ATZ in the liver and mediates a decrease in hepatic fibrosis. These results show that Flu can reduce the proteotoxicity of ATZ accumulation *in vivo* and, because it has been used safely in humans, this drug can be moved rapidly into trials for liver disease due to ATD. The results also provide further validation for drug discovery using *C. elegans* models that can be adapted to high-content drug screening platforms and used together with mammalian cell line and animal models.

## Introduction

Alpha-1-antitrypsin deficiency (ATD) has long been recognized as a genetic cause of liver disease in children. Recently it has become apparent that liver disease due to ATD may be even more significant in the adult population. The United Network of Organ Sharing reports ∼90–100 liver transplant procedures per year for ATD with 85–90% being done in adults (UNOS data, 2012, personal communication).

The liver disease of ATD is characterized by mostly fibrosis/cirrhosis with relatively mild inflammation and steatosis and it predisposes to hepatocellular carcinoma [1], [2]. The vast majority of these cases are caused by the classical form of ATD, homozygous for the *ATZ* allele. This allele bears a mis-sense mutation that is associated with misfolding and a tendency for the mutant polypeptide to polymerize and aggregate [3]. The mutant ATZ protein accumulates in the endoplasmic reticulum (ER) of hepatocytes, leading to cellular dysfunction by gain-of-toxic function mechanisms [3].

We have investigated the mechanisms by which accumulation of mutant ATZ in the ER is counteracted by endogenous protein degradation machinery and have found that the proteasomal and autophagic degradative systems both participate [4]. Retrograde translocation as a part of the ERAD pathway, ubiquitination and ultimately the proteasome probably participate in the degradation of soluble forms of ATZ in the ER at low levels of expression [4]–[6]. The autophagic system appears to play an important role in degradation of insoluble polymers/aggregates and perhaps also contributes to the fate of soluble forms of ATZ [5,7,and 8]. Autophagy appears to be particularly important when there are high levels of expression in model systems [8]. As shown by studies using the PiZ x GFP-LC3 mouse model of ATD, autophagy is specifically activated when ATZ expression leads to accumulation in the ER of liver cells *in vivo* [7]. There is also long-standing evidence for non-autophagic, non-proteasomal mechanisms contributing to intracellular degradation of ATZ, including a sortilin-mediated Golgi-to-lysosome pathway [9], and other as yet uncharacterized pathways are likely to be involved as well.

Because autophagy appears to play a key role in intracellular disposal of insoluble ATZ and because it is specifically activated when ATZ accumulates in the ER, we recently hypothesized that drugs which enhance the autophagic pathway could reduce the hepatocellular load of mutant ATZ and therein ameliorate its major proteotoxic sequella, hepatic fibrosis, in ATD. We found that a commonly used anticonvulsant and mood stabilizer, carbamazepine (CBZ), enhanced autophagic disposal of ATZ, reduced hepatic load of ATZ and hepatic fibrosis in the PiZ mouse model of ATD [10]. In a novel *C. elegans* model of ATD that robustly recapitulates the human disease, high-content screening of a relatively simple drug library discovered 3 drugs which reduce the cellular and organismal load of ATZ and also have potent autophagy enhancer activity [11]. Together, these findings provide powerful evidence for the concept that drugs which enhance proteostasis mechanisms, in this case autophagy, represent attractive candidates for chemoprophylaxis of liver disease in ATD.

One of the drugs that was identified by unbiased screening of the LOPAC drug library using the *C. elegans* model of ATD, fluphenazine, is a mood stabilizer that has been used safely in humans for many years. Here we show that it has potent autophagy enhancer activity and reduces the cellular load of ATZ in the *C. elegans* model, in mammalian cell line models and in the PiZ mouse model of ATD. Most importantly this drug also reduces hepatic fibrosis in the PiZ mouse. The study therefore provides a basis for considering clinical trials of this drug for patients with severe liver disease due to ATD.

## Materials and Methods

### Ethics Statement

This study was carried out in strict accordance with the recommendations in the Guide for the Care and Use of Laboratory Animals of the National Institutes of Health. The protocol was approved by the Institutional Animal Care and Use Committee (IACUC; permit number: 1009948) of the University of Pittsburgh. Surgery was performed under isoflurane anesthesia, and all efforts were made to minimize suffering.

### Materials

Rabbit anti-human AT antibody was purchased from DAKO (Santa Barbara, CA) and goat anti-human AT from Diasorin (Stillwater, MN). Antibody to GAPDH was purchased from US Biochemical. Living Colors®A.v. antibody was purchased from Clontech (Mountain View, CA). Antibody to LC3 was purchased from Novus Biologicals (Littleton, CO). Antibody to p62 was purchased from Cell Signaling Technology (Danvers, MA). Fluphenazine was purchased from Sigma and prepared as a stock solution of 5 mg/ml DMSO. Carbamazepine (CBZ) was purchased from Sigma and prepared in a stock solution of 25 mg/ml DMSO. Doxycycline was purchased from Sigma and prepared 1mg/ml in water. Flu and CBZ in sustained release pellets were manufactured by Innovative Research of American (Sarasota, FL).

### Cell Lines

The human epidermal HeLa cell line with doxycycline-regulated expression of ATZ (HTO/Z) has been described previously [12]. HTO/M, HTO/Saar and HTO/SaarZ are HeLa cell lines with doxycycline-regulated expression of wild type AT, AT Saar variant, and AT Saar Z variant respectively [12]. The CHOK1-Z cell line with stable constitutive expression of ATZ in Chinese hamster ovary cells has been previously described [13].

The HG2TONBZ#2 and HG2TONGZT#1 cell lines are derived from the human hepatoma cell line HepG2. The parent HepG2 cell line was engineered in each case for inducible (Tet-On) expression of ATZ-fluorophore chimeric proteins. For the HG2TONBZ#2 line the fluorophore CFP was engineered in-frame at the carboxyl terminus of ATZ. Expression of ATZ-CFP cannot be detected in the absence of doxycycline (dox) and increases profoundly within 6 hours when dox is added and the cellular properties of ATZ-CFP appear to recapitulate what is seen for ATZ in other genetically engineered cell lines. This includes alterations in the kinetics of secretion by pulse-chase analysis and accumulation in the insoluble fraction whereas a chimeric protein with CFP engineered at the carboxyl terminus of the nonpolymerogenic variant AT Saar does not appear at all in the insoluble fraction of cellular homogenates (K Covella, P Hale, DH Perlmutter, unpublished).

For the HG2TONGZT #1 cell line the fluorophore is GFP and engineered in frame at the amino terminus of the ATZ. Expression of ATZ is not detected in the absence of doxycycline (dox) but is induced and increases markedly in time- and dose-dependent fashion when dox is added. This cell line also recapitulates the know properties of ATZ, including intracellular accumulation and formation of insoluble polymers/aggregates, in contrast to 2 different HG2TONGMT cell lines with Tet-On inducible expression of wild type AT with GFP engineered into the amino terminus of wild type AT (K Covella, P Hale, DH Perlmutter, unpublished).

For experiments with Flu, the Tet-off inducible cell lines were cultured in the absence of doxycycline for at least 6 days to ensure expression of AT. The Tet-on inducible cell lines were cultured in the presence of doxycycline 200 ng/ml for 3 days for optimal expression. The cells were then subcultured into separate monolayers in fresh complete growth medium and incubated for 48 hours in the absence or presence of Flu or CBZ. Flu or CBZ was added to the growth medium. The duration of incubation with Flu was determined to be optimal at 48 hours based on experiments in which the duration was varied from 24 to 72 hours. Doses of Flu were initially based on previous studies of its effects in cell lines [14], [15]. After the incubation cells were homogenized and cell homogenates separated into insoluble and soluble fractions according to our previously established technique [13]. Samples of 10 µgs each were subjected to immunoblot analysis for AT and GAPDH.

### Transgenic Mice

PiZ mice that have been bred into the FVB/N background have been described previously [10]. Sections of liver tissue were stained with hematoxylin and eosin, PAS, PAS after diastase treatment, TUNEL, and Sirius Red using standard techniques [17], [18]. Sections of liver tissue were also stained with goat anti-human AT followed by donkey anti-goat Cy3 to detect AT-containing intracellular globules. The number of AT-containing globules was quantified blindly by counting cells in 6 microscopic fields of 10 different sections for each liver. The number of nuclei, as determined by Hoechst staining, was used to exclude the possibility that different numbers of cells were counted in liver sections from mice treated with Flu or CBZ as compared to controls. Sirius Red staining was also quantified by blindly analyzing 6 microscopic fields of 10 different sections for each liver using percent area stained red as determined by ImageJ software [10]. For quantification of TUNEL staining only cells with positive staining over the entire cell body were counted. The number of TUNEL+ hepatocytes per high power field (∼250 cells per high power field) was counted in 6 fields of 10 different sections for each liver. Hepatic hydroxyproline concentration was assayed as previously described (10).

For experiments involving drug administration in mice *in vivo*, doses of 1–10 mg/kg/day for Flu was based on previous studies of its biological effects in mice [14], [15]. The duration of 3 weeks was based on our experimental series with CBZ [10]. Flu and CBZ was delivered by oral gavage or slow-release subcutaneous pellets formulated by Innovative Research of America. Control mice were given DMSO by gavage or placebo pellets specifically designed for each of these drugs.

### Radioimmunoprecipitation, SDS-PAGE and Immunoblot Analysis

Biosynthetic labeling, pulse-chase labeling, immunoprecipitation and SDS-PAGE/fluorography for AT followed previously published protocols [12]. Radioactivity measured in TCA precipitates, using previous methods [10], did not show any effects of Flu on total protein synthesis or secretion (data not shown). For the pulse labeling experiments, HTO/Z cells were incubated for 48 hours in the absence or presence of Flu in several different concentrations and then subjected to labeling for 30 mins. The cell lysates were then examined by immunoprecipitation and the immunoprecipitates analyzed by SDS-PAGE/fluorography. For the pulse-chase experiments, HTO/Z cells were incubated for 48 hours in the absence of presence of Flu 0.1 nM and then pulse labeled for 60 mins. The cells were then washed and incubated in growth medium without tracer for several different time intervals to constitute the chase. Flu was included during the pulse and chase periods. The extracellular fluid and cell lysate samples were subjected to immunoprecipitation and the immunoprecipitates analyzed by SDS- PAGE/fluorography. All fluorograms were subjected to densitometry. The relative densitometric value of T0 is set at 100% and the remainder of the data set expressed as % of this control. The data are shown as mean +/− SD and the mean value at each time point is shown at the bottom of the figure.

For immunoblot analysis to detect AT or GAPDH, cells were lysed in 50 mM Tris-HCl, 150 mM NaCl, 1% NP-40, pH 8.0. Protein levels were quantified using the BCA protein assay (Pierce Biotechnology, Rockford, IL). 10 µg samples were loaded onto 10% precast gels. PVDF membranes were blocked in TBS, 0.5% Tween 20 (TBST), 5% milk and then incubated with primary antibody in 5% milk TBST solution. Horseradish peroxidase anti-goat Ig or anti-mouse Ig (Jackson Labs, Bar Harbor, ME) were used as secondary antibodies in TBST. Blots were visualized with Super Signal West Dura or West Femto from Pierce. For immunoblot on liver, the liver was snap frozen in liquid nitrogen and stored at −80°C. Liver was homogenized in 50 mM Tris-HCl pH 8.0, 150mM NaCl, 2 mM KCl, 2 mM MgCl2, 0.5% Triton X-100, 0.5% deoxycholic acid containing 0.1 mM phenylmethylsulfonic acid and complete protease inhibitor cocktail from Roche. Total protein concentration was measured by BCA assay (Pierce). Soluble and insoluble fractions were separated by centrifugation (14,000 rpm, 10 min, 4°C). The insoluble pellet was washed twice in 50 mM Tris-HCl (pH7.4, 150 mM NaCl) and resuspended in 50 mM Tris-HCl (pH6.8, 5% SDS, 10% glycerol). Equal amounts of total protein (1 ug) were loaded on 8% SDS-PAGE. After transfer to PVDF membrane, the blots were blocked in PBS-Tween20 containing 5% non-fat milk for 1 hr at RT, then goat-anti human AT antiserum (Diasorin, 1∶2500) was applied followed by three washes. Donkey anti-goat IgG-HRP (Santa Cruz, 1∶1,000,000) and West Dura (Pierce) was used for detection of AT. Anti-living color antibody was used to detect ATZ-CFP chimeric protein. The blots were also subjected to staining for GAPDH which is only in the soluble fraction to determine whether the soluble and insoluble fractions were separated appropriately and that loading was equivalent for the samples from the soluble fractions. This involved stripping (Pierce) and after the blocking step anti-mouse GAPDH (US Biologicals, 1∶10,000) and rabbit anti-mouse IgG-HRP (Jackson Labs, 1∶5000) were used to detect GAPDH. The blots were also stained with GelCode Blue (Pierce) so that loading of insoluble fractions could be assessed. Quantification was carried out by densitometric analysis using the ratio of ATZ to GAPDH for the soluble fractions and of ATZ to GelCode Blue-stained bands in the insoluble fraction.

Samples of 60 µgs each were subjected to immunoblot analysis for LC3 (16% precast gel) and p62 (10% gel) in mouse liver whole cell lysates [19]. Rabbit anti-LC3 (Novus, 1∶500) or rabbit anti-p62 (Cell Signaling, 1∶1000) was applied as primary antibody, anti-rabbit Ig (Jackson Labs, 1∶50,000) was used as secondary antibody in TBST.

For ELISA on mouse serum specimens, we first coated Nunc Maxisorp plates with goat anti-human AT (Bethyl), then blocked in PBS-Tween20 containing 5% nonfat milk. Serum samples were loaded into the wells in 1∶20,000 dilutions using purified human AT serial dilutions (1.56 to 100 ng/ml) as a standard. Rabbit-anti human AT (Dako) was used as capturing antibody, and goat anti-rabbit IgG-HRP (Dako) as secondary antibody. Protein levels were detected with OPD (Sigma).

### Real Time Quantitative PCR

Total RNA was extracted from PiZ mouse livers with TRIZOL (Life Technologies, Grand Island, NY). First strand cDNA (RT reaction) was synthesized from 2 µg of RNA using high capacity RNA-to-cDNA kit (Applied Biosystems, Foster City, CA) according to the manufacturer’s directions. A negative control was performed without enzyme (NRT reaction). RT and NRT reactions were also performed on 2 µg of commercially prepared liver RNA (Ambion, Austin, TX) to serve as the calibrator for the real time QPCRs. Each experimental sample was normalized to a nontransgenic control (fold change). For PCR, duplicate aliquots of the RT reaction and 1 aliquot of the NRT reaction served as templates for the target genes and the control gene β-glucuronidase (GusB). The probes and primers were obtained from Applied Biosystems (SERPINA1 assay ID: Hs01097800-m1; mouse GusB assay ID: Mm00446953**-**m1; human GusB assay ID: Hs9999908-m1). Real time reactions were run on an ABI7300 using the following cycling parameters: 95°C for 12 min, followed by 40 cycles of 95°C for 15 s and 60°C for 1 min. Differential gene expression was calculated by the ΔΔ*CT* calculation [16] The ΔΔ*CT* method controls for potential differences in efficiency of the RT, as well as the PCR, whereas calculations based on standard curves do not (ΔΔ*CT* = Δ*CT*(exp) - Δ*CT*(ctr) when Δ*CT* = Δ*CT*(gene) - Δ*CT*(norm)).

### *C. elegans* Strains and Culture Conditions

*C. elegans* strains, N2, VK1882 (*vkIs1882[nhx-2p::sGFP::ATZ;myo-2p::mCherry])* and VK1093 (*vkEx1093[nhx-2p::mCherry::lgg-1*]) were routinely cultured at 20°C on nematode growth medium (NGM) plates seeded with *E. coli* strain, OP50. Longevity experiments were performed NGM agar plates supplemented with DMSO or Flu to a final concentration of 50 µM. Kaplan-Meier curves were plotted and median survival times were calculated using *Prism*®, GraphPad.

### Imaging of Transgenic Animals using ArrayScan V^TI^

Quantitative analysis of animals expressing fluorescent transgenes was performed using the ArrayScan V^TI^ HCS Reader (Cellomics, ThermoFisher, Pittsburgh, PA, USA) fitted with a 2.5x objective and a 0.63x coupler as previously described [11]. Briefly, 35 L4 stage animals were placed into wells of 384-well plates containing DMSO or Flu to a final concentration of 50 µM. Worms were sorted using the COPAS Biosort to ensure that only animals of the similar age and fluorescence intensities were used for the experiment. Following incubation at 20°C for 24 hours, sodium azide was added to a final concentration of 50 µM to anesthetize the animals. Animals were then imaged on the ArrayScan V^TI^ (Thermo Scientific) and protein aggregates were quantified using the SpotDetector BioApplication (Thermo Scientific).

### Quantification of mCherry::LGG-1 Puncta

For microscopic image acquisition, approximately 12 worms were transferred to a 35 mm MatTek glass bottom culture dish (MatTek, Ashland, MA) containing 6 µl of 50 mM sodium azide. Confocal images were collected using a Leica TCS SP8 microscope and visualized, rendered and analyzed using Volocity Software (v6.11, Perkin Elmer). LGG-1 puncta were quantified using the Threshold Object Identification method in *Volocity.*

### Statistical Analysis

Students t-test was used for most comparisons but the Welch modified t-test was used to compare experimental groups that were not paired and did not assume equal variances. Kinetic and dose-response curves were analyzed by two-way ANOVA with the Bonferroni post-test using the Prism software application.

## Results

### Fluphenazine Mitigates the Proteotoxic Effects of ATZ Accumulation in vivo using the *C. elegans* Model

**Figure 1:**
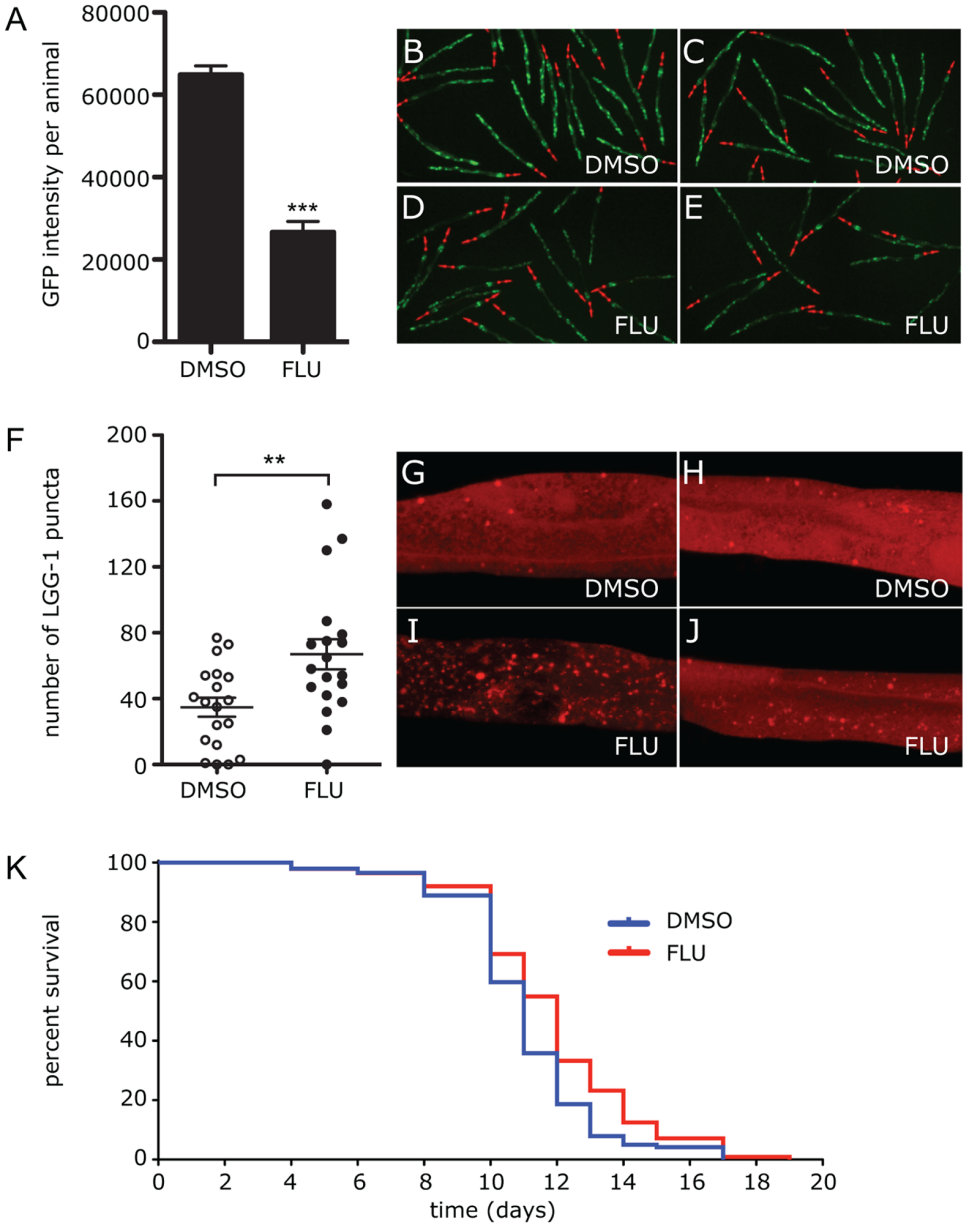
Effect of Flu treatment on clearance of ATZ and autophagy. Animals expressing sGFP::ATZ were treated with DMSO or 50 µM Flu for 24 h and GFP intensity measured using the Arrayscan V^TI^ (A). An average of three independent experiments with total n >300 animals per treatment is shown. Error bars represent SEM. Statistical significance was determined by using a Student’s t-test, ***P<0.001. Representative Arrayscan images of animals treated with DMSO (B,C) or 50 µM Flu (D,E). Effect of Flu treatment on LGG-1 puncta (F–J). Transgenic animals expressing mCherry::LGG-1 were treated with DMSO or Flu for 24 h and imaged using a Leica TCS SP8 microscope. LGG-1 puncta was quantified using Threshold Object Identification method in *Volocity* (Perkin Elmer, v5.4). Graph shows the average number of LGG-1 puncta in the posterior intestine of the animal (F). Statistical significance was determined using a Student’s t-test. **P<0.01. Representative confocal images of an mCherry::LGG-1 expressing animal treated with DMSO (G,H) or 50 µM Flu (I,J). Effect of Flu treatment on longevity of worms (K). The Kaplan-Meier graph showing the average lifespan of ATZ animals treated with DMSO (blue) or 50 µM Flu (red). Animals treated with 50 µM Flu had significantly (p<0.001) improved lifespan. Statistical significance was determined using Log-rank (Mantel-Cox) test. Data shown is an average of 3 experiments, n = 150 animals/treatment.

In a previous study we used a high-content small molecule screen of the *C. elegans* model and identified Flu as a drug which reduced accumulation of ATZ [11]. Here we sought to determine whether Flu also mitigates the proteotoxic effect of ATZ accumulation in this model. We used longevity because our studies have shown that ATZ expression specifically reduces longevity in *C. elegans* [SCP, GAS, DHP, unpublished]. A dose of 50 µM Flu was found to be ideal for longevity experiments. To ensure that this dose reduces ATZ accumulation we incubated animals for 24 hours with Flu 50 µM or DMSO in the same volume added to their feed. sGFP::ATZ accumulation was monitored by GFP fluorescence using the Arrayscan V^Ti,^ as previously described [11]. Flu mediated a reduction of GFP intensity that was close to 3-fold, p<0.001 (Figure 1A–E). To determine whether this dose enhances autophagy we carried out the same experiment using transgenic *C. elegans* expressing mCherry::LGG-1 (LC3)chimeric protein which labels autophagosomes with red fluorescence. The results show that LGG-1-positive puncta are significantly elevated (p<0.01 in the animals treated with Flu (Figure 1F–J). The increase in autophagosomes reached a magnitude of almost 2-fold, p<0.01 (Figure 1F Although the increase in LGG-1-positive puncta could be attributed to either activation of autophagy or decreased autophagosome clearance due to a block in autophagosome-lysosome fusion, the latter is unlikely because we have observed completely different results when *C. Elegans* is treated with Flu as compared to when autophagosome-lysosome fusion is inhibited in *C. elegans* by Rab 7 RNAi. In the former there is increased LGG-1-positive puncta and decreased ATZ levels (Fig 1A–J) whereas in the latter there are increased ATZ levels (data not shown).

To determine the effects of Flu on longevity, transgenic animals expressing sGFP::ATZ were incubated with 50 µM Flu or DMSO throughout their lifetime. In three separate experiments Flu was found to increase median survival time by an average of 25 hours (Table 1 and Figure 1K). This increase in longevity was significant (p<0.001), especially since the common laboratory strain only lives <20 days. Interestingly, this increase in survival was not observed when N2 control animals were treated with Flu indicating that Flu does not cause a general improvement in lifespan (Figure S1). Taken together, these data indicate that Flu mitigates at least one of the proteotoxic effects of ATZ accumulation *in vivo* at doses that reduce ATZ accumulation and increase autophagosomes.

**Table 1:** Effect of Fluphenazine on median survival times of sGFP:: ATZ animals.

Experiment	DMSO (hrs)	FLU50 (hrs)
1	500	526
2	527	551
3	505	530
Average	511	536*

The sGFP::ATZ worm strain was treated with Flu 50 µM or DMSO for determination of life-span. Results are presented as median survival time (hours) and were analyzed for statistical significance using Student’s t-test.

* p<0.05.

### Fluphenazine Reduces the Cellular Load of ATZ in Mammalian Cell Line Models of ATD

**Figure 2:**
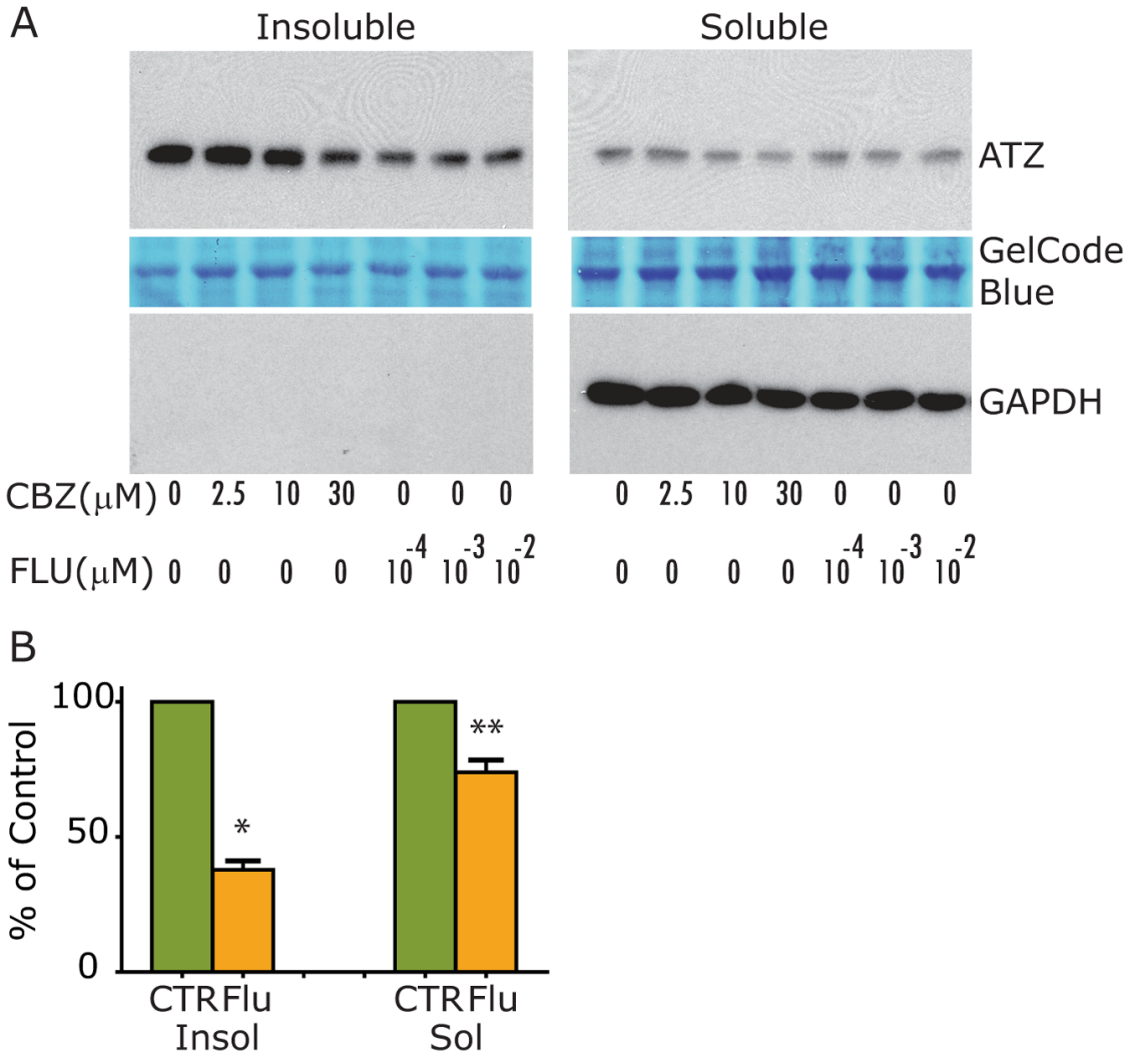
Effect of Flu on steady state levels of ATZ in genetically engineered HeLa HTO/Z cell line. Separate monolayers were incubated for 48 hours in the absence or presence of Flu or CBZ. Drug was added to the medium daily. Cells were then harvested, homogenized and the cell homogenates separated into soluble and insoluble fractions. The fractions were analyzed by immunoblot for AT (top) as well as loading controls, GelCode Blue (middle) and GAPDH (bottom). A, HTO/Z cell line expressing mutant ATZ; B, Densitometric analysis of 4 separate experiments in HTO/Z cell line. Mean +/− standard error is shown with error bars. Asterisks denote a statistically significant difference (p = 0.0029 for insoluble ATZ; p = 0.0292 for soluble ATZ).

**Figure 3:**
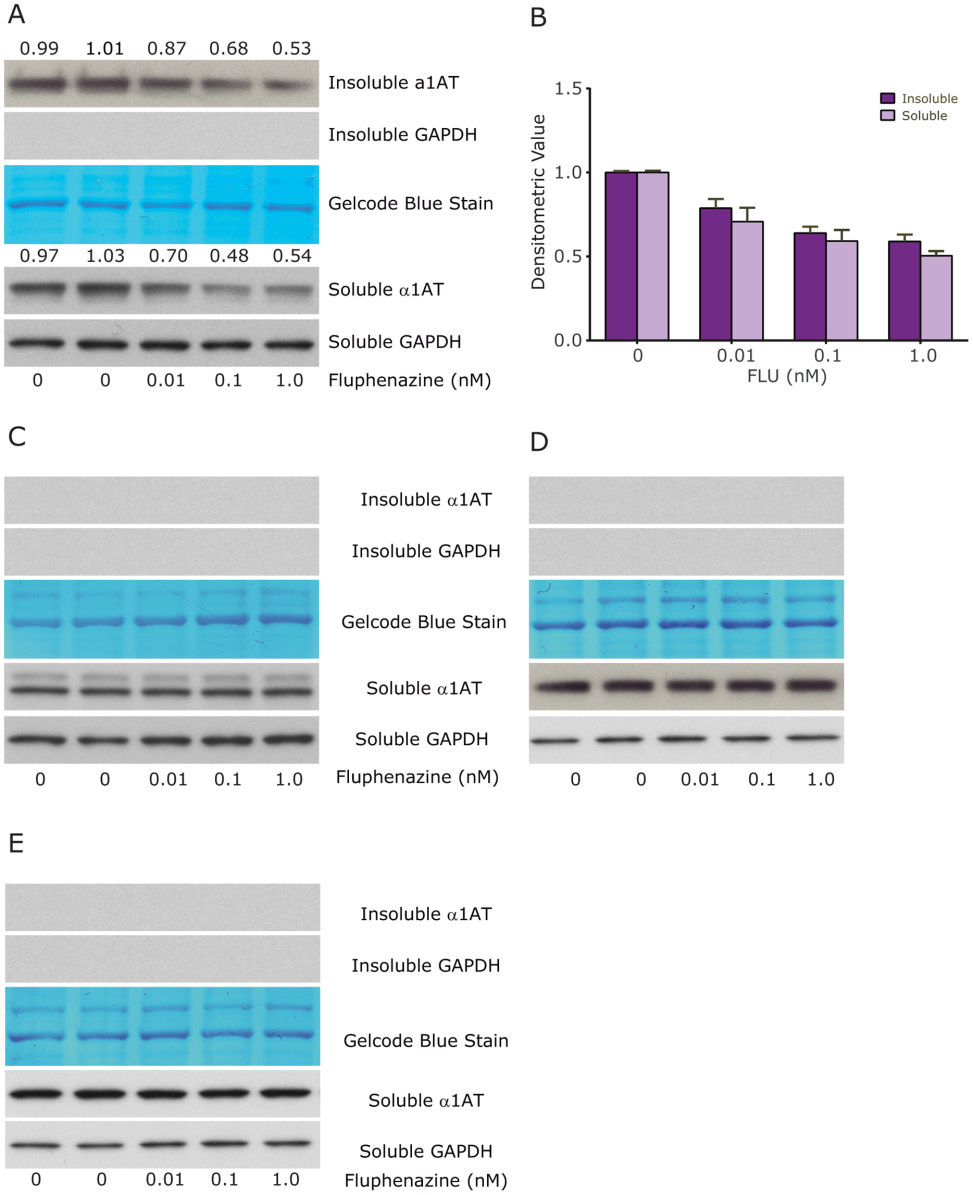
Effect of Flu on steady state levels of ATZ compared to wild type AT and other AT variants in genetically engineered HeLa cell lines. Exactly as in Figure 2. A, HTO/Z cell line expressing mutant ATZ; changes in relative ATZ levels as determined by densitometry is shown at the top of the panel; B, Dose-response relationship for the effect of Flu on soluble and insoluble ATZ levels in the HTO/Z cell line. These data represent mean +/− standard error from 4 separate experiments and the effect of dose is highly significant, p<0.0001 by two-way ANOVA; C, HTO/M cell line expressing wild type AT; D, HTO/Saar cell line expressing mutant AT Saar; E, HTO/Saar Z cell line expressing mutant AT Saar Z. In each case, GAPDH is used as a loading control for the soluble fraction and GelCode Blue stained bands are used as a loading control for the insoluble fraction.

The HTO/Z cell line was incubated for 48 hours in the absence or presence of CBZ or Flu and then the cells were homogenized for analysis of the cell homogenates by immunoblot (Fig 2). The results show that Flu reduces the steady state levels of ATZ in insoluble and soluble fractions (Fig 2A). However, the effect is significantly more potent on insoluble than soluble ATZ levels. It is first apparent at 0.1 nM. Indeed, the effect of Flu on insoluble ATZ is greater in magnitude and at lower doses than the effect of CBZ. Levels of GAPDH show that the differences in the soluble fraction are not due to loading and demonstrate the integrity of separating soluble from insoluble fractions of cell homogenate. Densitometric analysis of multiple experiments shows that the effect of 0.1 nM Flu on both insoluble and soluble ATZ levels is statistically significant (Fig 2B). The effect of Flu on ATZ levels in the HTO/Z cell line is also dose-dependent as shown best by the densitometric analysis of ATZ levels in Fig 3B. Flu, used at the same concentrations and time interval, did not affect steady state levels of wild type AT in the HTO/M cell line (Fig 3B), levels of mutant AT Saar in the HTO/Saar cell line (Fig 3C) or levels of mutant AT Saar Z in the HTO/SaarZ cell line (Fig. 3D).

**Figure 4:**
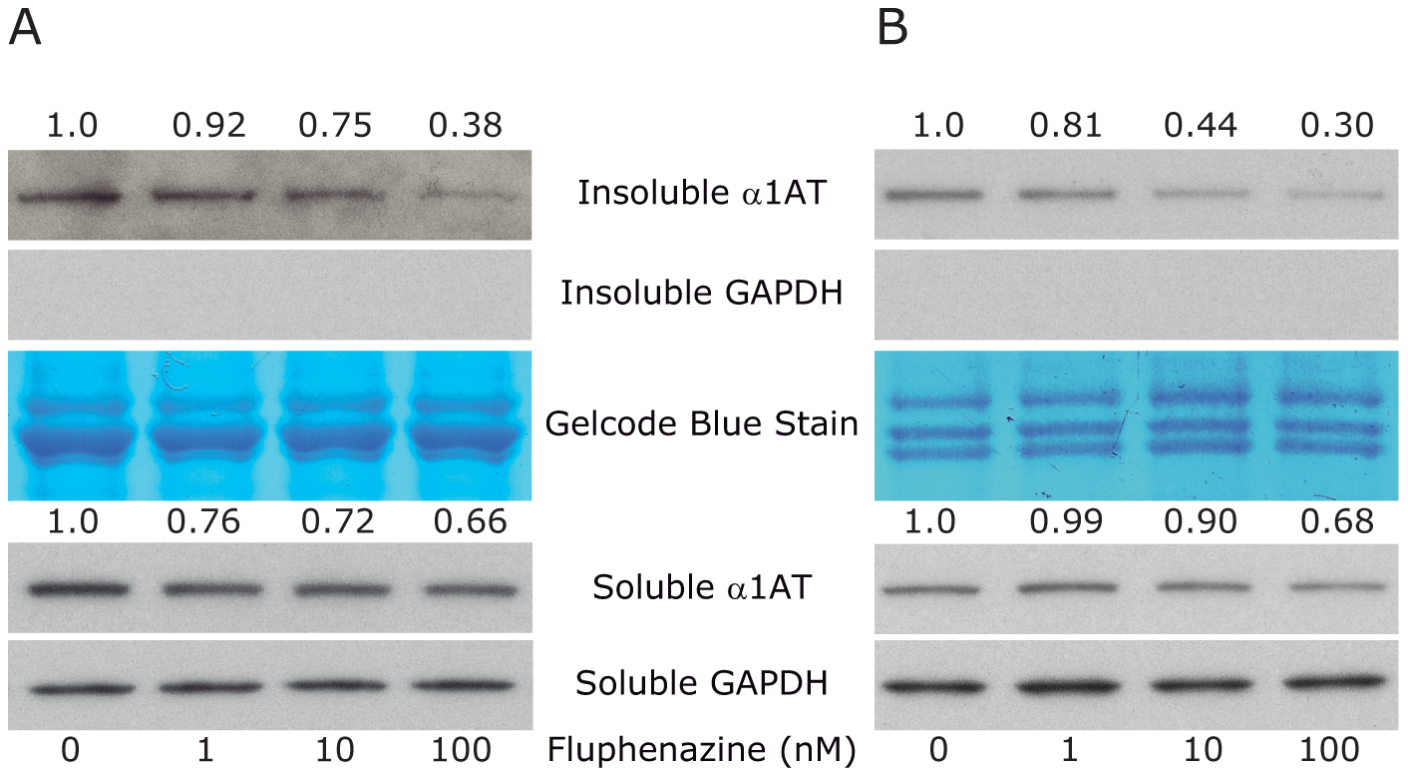
Effect of Flu on steady state levels of ATZ in other genetically engineered cell line models of ATD. Two different cell line models were analyzed for the effect of Flu exactly as described in Figure 2. A, HG2TONBZ#2; B, HG2TONGZT#1. In each case levels of ATZ and GAPDH are shown. GelCode blue stained bands are also shown as a control for the insoluble fractions. The change in level of ATZ as determined by densitometry is shown at the top of the panels.

To exclude the possibility that the effect of Flu was peculiar to the HTO/Z cell line we tested it in several other cell lines. Using 2 new cell lines, HG2TONBZ#2, which has inducible expression of an ATZ-CFP chimeric protein, and HG2TONGZT#1, a human hepatoma cell line with inducible expression of a GFP-ATZ chimeric protein, we found that Flu reduced the cellular load of ATZ (Fig 4). However, in these cell lines the effect of Flu required higher doses, 10–100 nM, and the drug had an even lesser effect on soluble ATZ levels than observed in the HeLa-based HTO/Z model system. Flu also mediated a reduction in cellular ATZ load in a CHO cell line with constitutive expression of ATZ (data not shown). Together these results indicate that the effect of Flu on intracellular ATZ accumulation is apparent in multiple mammalian cell lines. Elucidation of the mechanism of action will be necessary to determine why different doses of Flu are required for effects in the HepG2- compared to the HeLa-based cell line model.

### Effect of Fluphenazine on the Kinetics of Degradation and Secretion of ATZ in Cell Line Models of ATD

**Figure 5:**
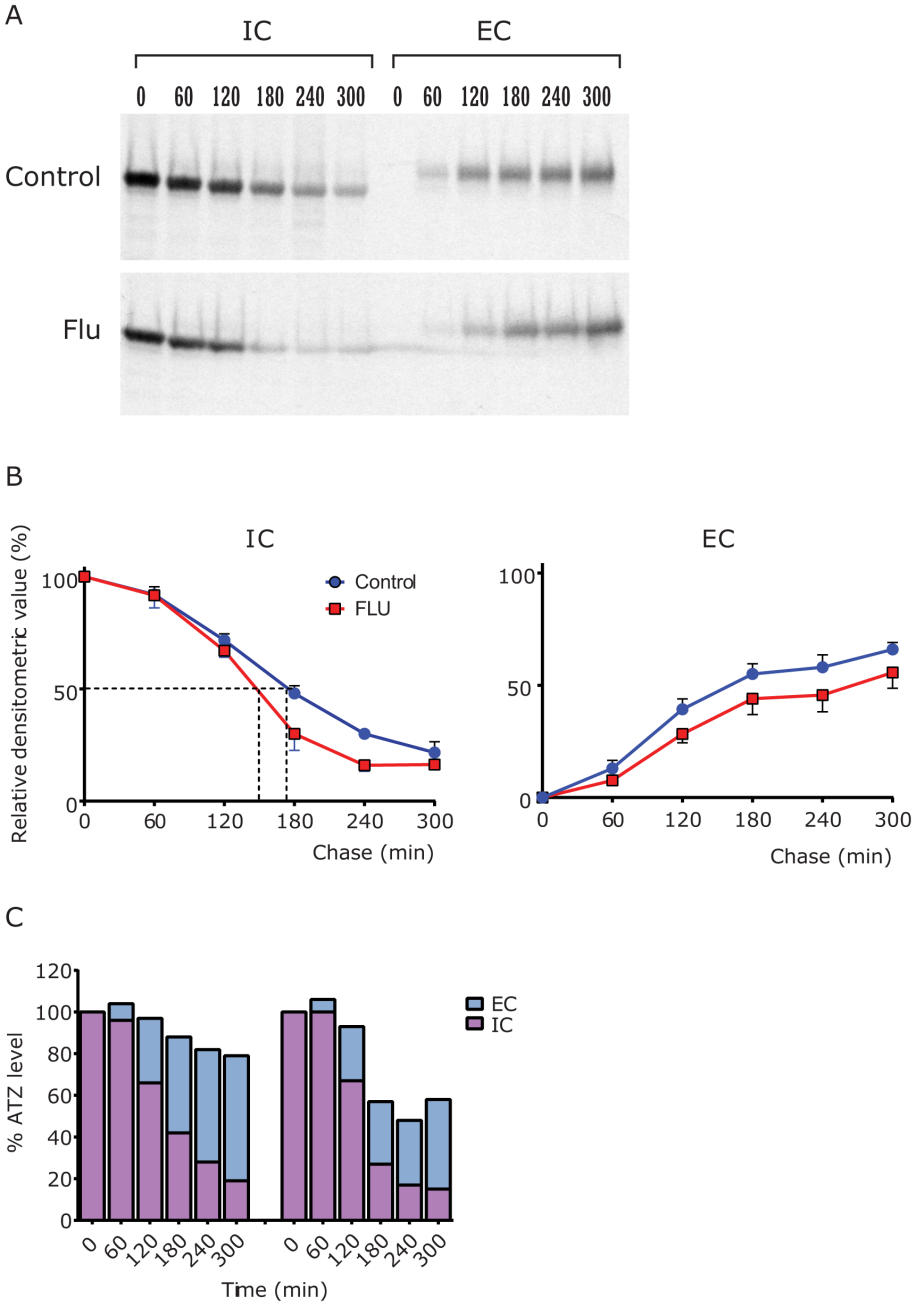
Effect of Flu on kinetics of secretion of ATZ in the HTO/Z cell line. Separate monolayers were incubated for 48 hours in the absence or presence of Flu (0.1 nM) and then were subjected to pulse radiolabeling for 60 mins. The monolayers were rinsed vigorously and then subjected to chase in medium with excess unlabeled methionine for time periods up to 240 mins. The extracellular fluid (EC) and cell lysates (IC) were analyzed by immunoprecipitation for AT followed by SDS-PAGE/fluorography. A, Fluorograms of control (top) and Flu-treated cells. Chase time points are shown at the top. B, Densitometric analysis of kinetics. Disappearance of ATZ from IC compartment is shown on the left and appearance in EC is shown on the right for n = 3 experiments. Mean +/−SEM is shown for each time point with error bars. The IC disappearance is increased significantly (p = 0.0012) and the EC appearance is decreased significantly (p = 0.0033), using the matched ANOVA in GraphPad. The half-time for disappearance is shown with dashed lines, 180 mins for control and 140 mins for Flu-treated cells. C, Densitometric analysis of ATZ fate. Representative fluorographic images were subjected to densitometric scanning and relative ATZ levels in intracellular and extracellular compartments are shown together for each time point. The relative densitometric intensity of the ATZ band at T0 IC is set at 100% and every other band is compared to that. The results for control are shown at left and for Flu on the right. This analysis shows loss of ATZ in the Flu-treated cells that can only be accounted for by increased degradation.

The HTO/Z cell line was subjected to pulse-chase radiolabeling analysis after incubation for 48 hours in the absence or presence of Flu 0.1 nM (Fig 5A). The results show that ATZ disappears earlier in cells treated with Flu as compared to control. Lesser ATZ is apparent at the later time points of the chase period. There is no difference in the time of appearance of ATZ in the extracellular fluid compared treated to untreated cells but lesser amounts of ATZ are present in EC of treated cells compared to control cells. These results are most consistent with an effect of the drug that exclusively increases the rate of intracellular degradation. Quantitative analysis by densitometry on multiple experiments (Fig 5B) shows that there is more rapid disappearance in the presence of Flu with a half-time for ATZ disappearance of 140 mins in treated cells versus 180 mins in control cells (22.2% increase in rate of degradation, p = 0.0012) and lesser amounts of ATZ in the EC, especially later in the chase period (120 to 300 mins, p = 0.0033). Applying a different type of quantitative analysis in which the amount of AT-specific radioactivity in the IC and EC is combined at each time point (Fig 5C), the selective effect of Flu on degradation is evidenced by the reduction in ATZ-specific radioactivity in Flu-treated cells during the chase.

### Fluphenazine Reduces Hepatic ATZ Load and Hepatic Fibrosis in the PiZ Mouse Model of ATD

**Figure 6:**
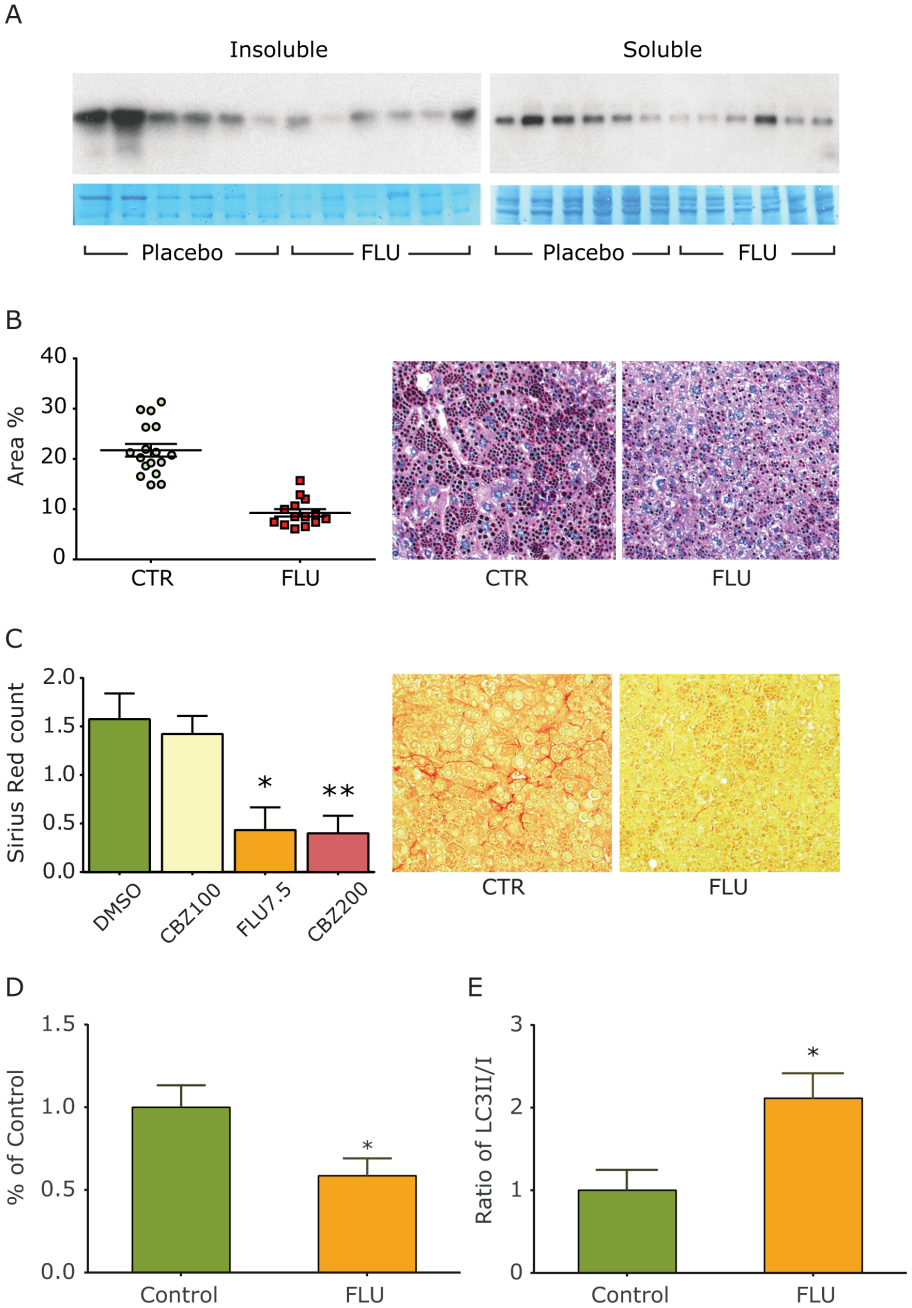
Effect of Flu on hepatic ATZ accumulation and hepatic fibrosis in the PiZ mouse model. At 3 months of age a sustained release pellet containing Flu, CBZ or placebo was inserted subcutaneously into PiZ mice. The pellets contained enough Flu to deliver 7.5/kg/d or CBZ to deliver 100 mg/kg/day or 200 mg/kg/day on the basis of the average weight of 3-mos old PiZ mice. At the end of 3 weeks, mice were sacrificed and the liver analyzed by immunoblot for AT (A), PAS/diastase staining (B), Sirius Red staining (C), immunoblot/densitometric analysis for p62 levels (D) and immunoblot/densitometric analysis for the LC3-II/I ratio (E). The immunoblot in Fig 6A shows ATZ levels at the top and staining with Gel Code Blue as a control at the bottom. In each case a single sample from the liver of 6 control and 6 Flu-treated PiZ mice is analyzed. The statistical analysis in panels (B) and (C) was carried out by Image J software determining percent area stained by PAS (B) and Sirius Red (C) in 6 microscopic fields from 10 sections of each liver specimen. The asterisks in panel (C) denote a statistically significant difference, p = 0.0105 for Flu and p = 0.0028 for CBZ. The asterisk in panel (D) denotes a statistically significant different decrease in p62 levels, p = 0.0354 and the asterisk in panel (E) denotes a statistically significant increase in LC3II/I ratio, p = 0.0075 in the livers of Flu-treated versus control mice (n = 6 each).

Our results indicate that Flu can reduce ATZ load *in vivo* using the *C. elegans* model but here we sought to determine if the drug can reduce ATZ levels and the major clinical sequella, fibrosis, in the PiZ mouse model. PiZ mice were treated with Flu 7.5 mg/kg/day or placebo by daily oral gavage or with sustained release pellets inserted subcutaneously. In Fig. 6A, the results show reduced hepatic levels of ATZ in mice treated with Flu. The steady state levels of insoluble ATZ are significantly reduced by a magnitude of 2.1-fold (p = 0.021). There was a trend toward lower levels of soluble ATZ but this trend did not reach statistical significance. There was no change in human ATZ mRNA levels in the liver of Flu-treated PiZ mice (data not shown). Most importantly there was significant reduction in ATZ globules as determined by PAS/diastase staining, p<0.0001 (Fig 6B) and by immunofluorescent staining for ATZ (data not shown). Flu also mediated a significant decrease in fibrosis as determined by Sirius red staining, p = 0.0105 for Flu (Fig 6C). The reduction in fibrosis was similar to the effect of CBZ (p = 0.0028). Hepatic hydroxyproline content was also significantly reduced by Flu (control 2.557+/−0.194 vs. Flu 1.868+/−0.186 µg/gm liver tissue, p = 0.0307). Although there was a trend towards reduced TUNEL+ hepatocytes in Flu-treated PiZ mice this difference did not reach statistical significance (p = 0.0741). There was no additive or synergistic effects of Flu and CBZ administered together (data not shown). Flu administration had no effect on serum levels of human AT using ELISA (data not shown). Doses of Flu below 7.5 mg/kg/day did not reduce ATZ levels, ATZ-containing globules or fibrosis (data not shown). Flu-treated PiZ mice had significantly reduced levels of p62 (Figure 6D) and increased LC3-II:LC3-I ratio (Figure 6E) in the liver compared to untreated PiZ mice. Together, these data indicate that Flu reduces hepatic ATZ load and fibrosis and these effects are associated with increased markers of hepatic autophagy in the PiZ mouse model of ATD *in vivo*.

## Discussion

A series of studies have shown that autophagy plays a critical role in intracellular disposal of the mutant ATZ molecule that aberrantly accumulates in liver cells and is responsible for hepatic proteotoxicity in the classical form of ATD [4]. Furthermore, we recently found a drug which enhances autophagy, CBZ, can reduce the hepatic load of ATZ and hepatic fibrosis in the PiZ mouse model of ATD [10]. In the current study we show that another drug which has the property of enhancing the autophagic pathway, Flu, reduces ATZ accumulation in a *C. elegans* model of ATD, reduces steady state levels of ATZ in 4 different mammalian cell line models of ATD and reduces hepatic ATZ accumulation in the PiZ mouse model of ATD. Most importantly, administration of this drug to PiZ mice reduces hepatic fibrosis, the most important toxic sequellae of ATZ accumulation in the liver. Like CBZ, Flu acts solely on the intracellular degradation of ATZ, with no apparent effect on synthesis or secretion of this mutant protein and it has no effect on wild type AT or on nonpolymerogenic AT variants. In contrast to CBZ which mediates decreases in both insoluble and soluble ATZ, Flu appears to act more robustly on insoluble ATZ. One might use this data to conclude that Flu acts solely on autophagic disposal of ATZ whereas CBZ acts on both autophagic and non-autophagic mechanisms for disposal of ATZ. However, it is still not clear how the various forms of ATZ that accumulate in the ER, including soluble monomers, soluble polymers and insoluble polymers/aggregates are segregated into the various pathways for disposal which include autophagic, proteasomal and non-autophagic, non proteasomal mechanisms.

Several results provide evidence that Flu enhances autophagy *in vivo*. In addition to increased LC3 II:I ratio and decreased p62 levels in the liver of the PiZ mouse after Flu administration, Flu mediated an increase in LGG-1-positive puncta in the *C. elegans* model *in vivo*. Although the latter result could be attributed to an effect of Flu that led to increased autophagosomes because of decreased autophagosome-lysosome fusion, we found that inhibition of autophagosome-lysosome fusion by Rab7 RNAi has effects that were completely opposite of those of Flu. When *C. elegans* was treated with Rab7 RNAi there were increased ATZ levels.

The results of this study provide a validation for the *C. Elegans* model of ATD and automated high-content drug screening platform recently developed using this model [11]. Flu was originally identified as a potential therapeutic compound by completely unbiased screening of the LOPAC drug library. The drug was then shown to have a reproducible dose-dependent effect on ATZ load and proteotoxicity when moved sequentially from the *C. elegans* model to a mammalian cell line and finally to a transgenic mouse model of ATD. This means that the *C. elegans* model/screening platform can be used effectively and robustly to identify therapeutic drug candidates and furthermore implies the extraordinary similarity in the cell biology and pharmacology of ATZ accumulation in worm and mammalian cells. Thus, we should see major advances in identification of therapeutic drugs and genetic modifiers of ATD as well as further understanding of mechanisms by which mutant ATZ elicits proteotoxic effects using this relatively simple and inexpensive model.

This study also provides further evidence for the application of drugs with autophagy enhancer activity to therapeutics for ATD. Previously we found that CBZ enhanced autophagic degradation of mutant ATZ and that this mechanism led to a reduction in hepatic fibrosis in the PiZ mouse model of ATD, presumably by reducing the proteotoxic effects of ATZ accumulation [10]. Subsequently we found that 3 hit compounds from our initial high-content screen of the *C. elegans* model have the property of enhancing autophagy [11]. Because the screening platform is set up in a way that it could identify drugs which work on cellular ATZ load by any possible mechanism, including decreased synthesis, increased secretion or increased degradation by non-autophagic mechanisms, we suspect that there is something particularly effective about the autophagy enhancer mechanism of drug action. In high-throughput screens for drugs which enhance autophagic degradation of another aggregation-prone protein, huntingtin, that have been carried out by 2 different laboratories using different mammalian cell line models [20], [21], drugs in the phenothiazine family have been particularly prominent among the hit compounds. Indeed, pimozide was a hit compound in one of these one of these screens [20] as it was in our screen [11] and the phenothiazine structure was used to develop a pharmacophore model for virtual screening to identify additional novel drugs for Huntington’s disease [21]. Flu is also a member of the phenothiazine drug family with autophagy enhancer activity and herein is shown to have a putative therapeutic effect on hepatic fibrosis due to ATD. It is also important to note that CBZ has structural similarities to the tricyclic and phenothiazine mood stabilizing drugs. Together, these observations lead us to conclude that the autophagy enhancer class of drugs and the phenothiazine structure constitute excellent leads for further efforts at drug discovery for ATD.

Although it is known that Flu and other members of the phenothiazine drug family act on dopamine D2 receptors, there is relatively little known about the effect of these drugs at the cellular/biochemical level. Older literature mentions effects of Flu and other phenothiazines on calcium stimulus-response coupling [22], [23], properties that they could hold in common with CBZ and the autophagic response is known to be regulated by cellular calcium [24]. It is also worth noting that there is an appealing teleological basis for the efficacy of drugs which enhance degradation of cytotoxic proteins as compared to nucleic acid-based therapeutics currently being considered to target production of cytotoxic proteins. Drugs which target degradation are acting at the step most proximal to the proteotoxicity and therefore may not need to be as effective as drugs which target much earlier steps and this may be particularly important for ATD because endogenous AT is produced at such high levels by the liver.

Because Flu is an FDA-approved drug with a well-established history of safe use, it is a good candidate for clinical trials to reduce proteotoxicity in ATD. Relatively high doses of Flu were required for beneficial effects in PiZ mice. The dose of 7.5 mg/kg/day in mice compares to 2.5–10 mg/day as the initial starting dose in humans, going up to 40 mg/day (equivalent of ∼0.57 mg/kg/day in a 70 kg man). This difference is very similar to what we observed for CBZ with doses between 100–200 mg/kg/day required in mice as compared to 10–20 mg/kg/day used in humans [10]. It is as yet unclear whether such differences will limit the effectiveness of these drugs in humans or will be explained by the known 10- to 20-fold difference in dose of drugs required because of the high surface area to body weight ratio in rodents compared to humans. Recently Flu was found to decrease steady state levels of aggregation-prone prion proteins in cell line and mouse models [25]. Although autophagy or other degradation pathways were not investigated, the results could easily be explained by enhanced autophagic disposal. These observations therefore provide a basis for further studies of Flu as a potential therapeutic for ATD as well as other diseases caused by aggregation-prone proteins.

## Supporting Information

Figure S1 **Effect of Flu treatment on longevity of N2 controls.** As a control for the ATZ longevity on Flu, the lifespan of N2 animals was assessed upon treatment with DMSO (blue) or 50 µM Flu (red). Animals treated with 50 µM Flu had a modest but statistically significantly (p<0.05) decrease in lifespan. Statistical significance was determined using the Logrank (Mantel-Cox) test. Data shown is an average of 3 experiments, n = 150 animals/treatment
(TIF)

## References

[1] Perlmutter DH, Silverman GA (2011) Hepatic fibrosis and carcinogenesis in α1-antitrypsin deficiency: A prototype for chronic tissue damage in gain-of-function disorders. Cold Spring Harb. Perspect. Biol. 3: 181–194. 10.1101/cshperspect.a005801 PMC3039936 21421920

[2] Perlmutter DH, Brodsky JL, Balistreri WF, Trapnell BC (2007) Molecular pathogenesis of alpha-1-antitrypsin deficiency-associated liver disease: A meeting review. Hepatology 45: 1313–1323. 17464974 10.1002/hep.21628

[3] Maurice N, Perlmutter DH (2012) Novel treatment strategies for liver disease due to α1-antitrypsin deficiency. Clin. Transl. Sci. 5: 289–294. 10.1111/j.1752-8062.2011.00363.x PMC3982223 22686209

[4] Perlmutter DH (2010) Alpha-1-antitrypsin deficiency: Importance of proteasomal and autophagic degradative pathways in disposal of liver disease-associated protein aggregates. Annu. Rev. Med. 62: 333–345. 10.1146/annurev-med-042409-151920 20707674

[5] Qu D, Teckman JH, Omura S, Perlmutter DH (1996) Degradation of a mutant secretory protein, α1-antitrypsin Z, in the endoplasmic reticulum requires proteasome activity. J. Biol. Chem. 271: 22791–22795. 10.1074/jbc.271.37.22791 8798455

[6] Werner ED, Brodsky JL, McCracken AA (1996) Proteasome-dependent endoplasmic reticulum-associated protein degradation: An unconventional route to a familiar fate. Proc. Natl. Acad. Sci. USA 93: 13797–13801. 10.1073/pnas.93.24.13797 PMC19430 8943015

[7] Kamimoto T, Shoji S, Hidvegi T, Mizushima N, Umebayashi K, et al (2006) Intracellular inclusions containing mutant α1-antitrypsin Z are propagated in the absence of autophagic activity. J. Biol. Chem. 281: 4467–4476. 10.1074/jbc.M509409200 16365039

[8] Kruse KB, Brodsky JL, McCracke AA (2006) Characterization of an ERAD gene as VPS30/ATG6 reveals two alternative and functionally distinct protein quality control pathways: One for soluble Z variant of human alpha-1-proteinase inhibitor (A1PiZ) and another for aggregates of A1PiZ. Mol. Biol. Cell 17: 203–212. 10.1091/mbc.E04-09-0779 PMC1345659 16267277

[9] Gelling CL, Dawes IW, Perlmutter DH, Fisher EA, Brodsky JL (2012) The endosomal protein sorting receptor sortilin has a role in trafficking alpha-1 antitrypsin. Genetics 192: 889–903. 22923381 10.1534/genetics.112.143487 PMC3522165

[10] Hidvegi T, Ewing M, Hale P, Dippold C, Beckett C, et al (2010) An autophagy-enhancing drug promotes degradation of mutant α1-antitrypsin Z and reduces hepatic fibrosis. Science 329: 229–232. 20522742 10.1126/science.1190354

[11] Gosai SJ, Kwak JH, Luk CJ, Long OS, King DE, et al (2010) Automated high-content live animal drug screening using C. elegans expressing the aggregation prone serpin α1-antitrypsin Z. PLoS One. 5: e15460. 10.1371/journal.pone.0015460 PMC2980495 21103396

[12] Hidvegi T, Schmidt BZ, Hale P, Perlmutter DH (2005) Accumulation of mutant α1-antitrypsin Z in the ER activates caspases-4 and -12, NFκB and BAP31 but not the unfolded protein response. J. Biol. Chem. 280: 39002–39015. 10.1074/jbc.M508652200 16183649

[13] Schmidt BZ, Perlmutter DH (2005) Grp78, Grp94 and Grp170 interact with α1-antitrypsin mutants that are retained in the endoplasmic reticulum. Am. J. Physiol. 289: G444–G455. 10.1152/ajpgi.00237.2004 15845869

[14] Bisson WH, Cheltsov AV, Bruey-Sedano N, Lin B, Chen J, et al (2007) Discovery of antiandrogen activity of nonsteroidal scaffolds of marketed drugs. Proc. Natl. Acad. Sci. USA 104: 11927–11932. 10.1073/pnas.0609752104 PMC1924583 17606915

[15] DePreter K, DeBrouwer S, Van Maerken T, Pattyn F, Schramm A, et al (2009) Meta-mining of neuroblastoma and neuroblast gene expression profiles reveals candidate therapeutic compounds. Clin. Cancer. Res. 15: 3690–3696. 10.1158/1078-0432.CCR-08-2699 19435837

[16] Livak KJ, Schmittgen TD (2001) Analysis of relative gene expression data using real-time quantitative PCR and the 2^−ΔΔ^C_T_ method. Methods 25: 402–408. 11846609 10.1006/meth.2001.1262

[17] Paranjpe S, Bowen WC, Bell AW, Nejak-Bowen K, Luo JH, et al (2007) Cell cycle effects resulting from inhibition of hepatocyte growth factor and its receptor c-Met in regenerating rat livers by RNA interference. Hepatology 45: 1471–1477. 17427161 10.1002/hep.21570 PMC2632963

[18] Osterreicher CH, Taura K, DeMinicis S, Seki E, Penz-Osterreicher M, et al (2009) Angiotensin-converting-enzyme 2 inhibits liver fibrosis in mice. Hepatology 50: 929–938. 19650157 10.1002/hep.23104 PMC4734904

[19] Mizushima N, Yoshimura T (2007) How to interpret LC3 immunoblotting. Autophagy 3: 542–545. 17611390 10.4161/auto.4600

[20] Zhang L, Yu J, Pa H, Hu P, Hao Y, et al (2007) Small molecule regulators of autophagy identified by an image-based high-throughput screen. Proc. Natl. Acad. Sci. USA 104: 19023–19028. 10.1073/pnas.0709695104 PMC2141901 18024584

[21] Tsvetkov AS, Miller J, Arrasate M, Wong JS, Pleiss MA, et al (2010) A small-molecule scaffold induces autophagy in primary neurons and protects against toxicity in a Huntington disease model. Proc. Natl. Acad. Sci. USA 107: 16982–16987. 10.1073/pnas.1004498107 PMC2947884 20833817

[22] Polya GM, Davies JR, Micucci V (1983) Properties of a calmodulin-activated Ca (2+)-dependent protein kinase from wheat germ. Biochim. Biophys. Acta. 76: 1–12. 10.1016/0304-4165(83)90355-0 6639959

[23] Kremer MS, Kenyon JL, Ito K, Sutko JL (1985) Phenothiazine suppression of transient depolarization in rabbit ventricular cells. Am. J. Physiol. 248: H291–296. 10.1152/ajpheart.1985.248.2.H291 3970229

[24] Decuypere JP, Bultynck G, Parys JP (2011) A dual role for Ca (2+) in autophagy regulation. Cell Calcium 50: 242–250. 21571367 10.1016/j.ceca.2011.04.001

[25] Chung E, Prelli F, Dealler S, Lee WS, Chang Y-T, et al (2011) Styryl-based and tricyclic compounds as potential anti-prion agents. PLoS One 6: e24844. 21931860 10.1371/journal.pone.0024844 PMC3172287

