# Supplementary figures and images for: Fluphenazine Reduces Proteotoxicity in C. elegans and Mammalian Models of Alpha-1-Antitrypsin Deficiency
Source: PLoS One. 2014 Jan 31;9(1):e87260. doi: 10.1371/journal.pone.0087260 (PMC3909079; doi:10.1371/journal.pone.0087260)

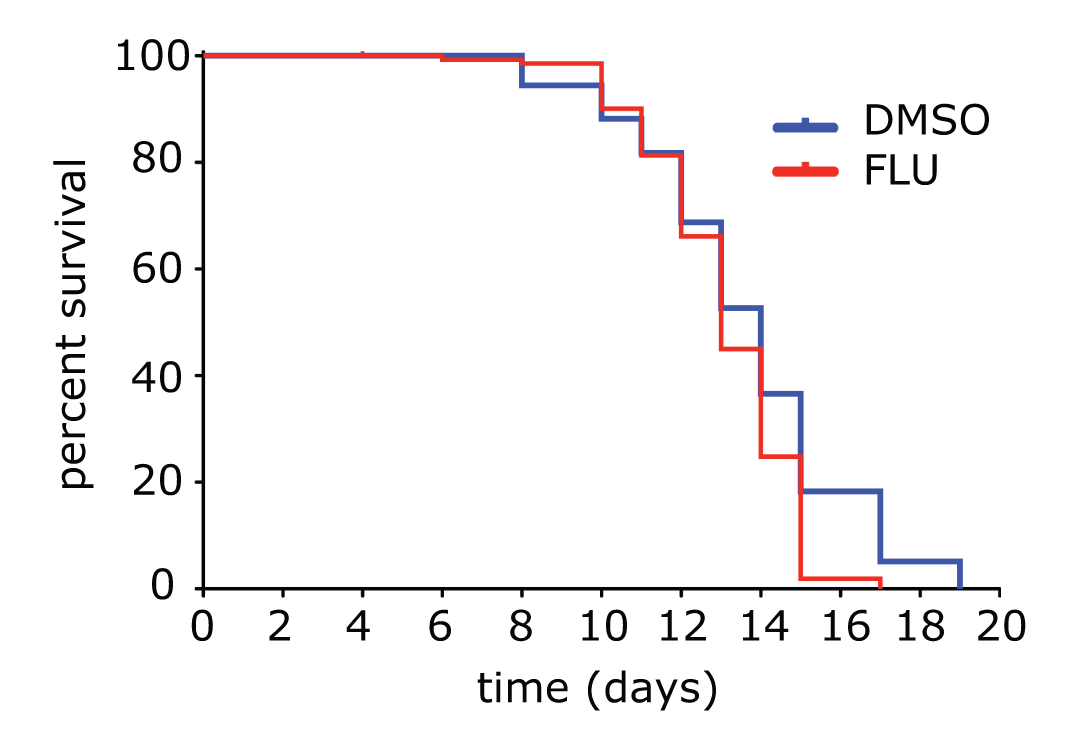

Supplement: Figure S1 — Effect of Flu treatment on longevity of N2 controls. As a control for the ATZ longevity on Flu, the lifespan of N2 animals was assessed upon treatment with DMSO (blue) or 50 µM Flu (red). Animals treated with 50 µM Flu had a modest but statistically significantly (p<0.05) decrease in lifespan. Statistical significance was determined using the Logrank (Mantel-Cox) test. Data shown is an average of 3 experiments, n = 150 animals/treatment (TIF) [file pone.0087260.s001.tif]
